# Associations between information and communication technology use and frailty in community-dwelling old-old adults: results from the ILSA-J

**DOI:** 10.1007/s41999-024-00979-y

**Published:** 2024-05-09

**Authors:** Daijo Shiratsuchi, Hyuma Makizako, Shoma Akaida, Mana Tateishi, Hirohiko Hirano, Katsuya Iijima, Minoru Yamada, Narumi Kojima, Shuichi Obuchi, Yoshinori Fujiwara, Hiroshi Murayama, Yukiko Nishita, Seungwon Jeong, Rei Otsuka, Takumi Abe, Takao Suzuki

**Affiliations:** 1 Department of Physical Therapy, Faculty of Medicine, School of Health Sciences, Kagoshima University, 8-35-1 Sakuragaoka, Kagoshima, 890-8544 Japan; 2 Research Team for Promoting Independence and Mental Health, Tokyo Metropolitan Institute for Geriatrics and Gerontology, Tokyo, Japan; 3 Institute of Gerontology, The University of Tokyo, Tokyo, Japan; 4 Institute for Future Initiatives, The University of Tokyo, Tokyo, Japan; 5 Faculty of Human Sciences, University of Tsukuba, Tokyo, Japan; 6 Research Team for Human Care, Tokyo Metropolitan Institute for Geriatrics and Gerontology, Tokyo, Japan; 7 Research Team for Social Participation and Healthy Aging, Tokyo Metropolitan Institute for Geriatrics and Gerontology, Tokyo, Japan; 8 Department of Epidemiology of Aging, National Center for Geriatrics and Gerontology, Aichi, Japan; 9 Department of Community Welfare, Niimi University, Okayama, Japan; 10 Institute of Gerontology, J.F. Oberlin University, Tokyo, Japan

**Keywords:** Frail, Information technology, Older people, Protective factor

## Abstract

**Objectives:**

Frailty is a significant cause of adverse health events including long-term care and hospitalization. Although information and communication technology (ICT) has become an integral part of modern life, it remains unclear whether ICT use is associated with frailty.

**Design:**

A cross-sectional study (Integrated Longitudinal Studies on Aging in Japan, ILSA-J).

**Setting and participants:**

Aged 75 and older data from the ILSA-J in 2017 (*n* = 2893).

**Methods:**

ICT use was measured using the technology usage sub-items of the Japan Science and Technology Agency Index of Competence. Specifically, the use of mobile phones, ATMs, DVD players, and sending e-mails were rated as “yes” (able to do) or “no” (unable to do), with the first quintile (≤1 point) defined as ICT non-users. Frailty was assessed using the Japanese version of the Cardiovascular Health Study criteria based on the phenotype model (e.g., weight loss, slowness, weakness, exhaustion, and low activity). Further, multivariate logistic regression analysis analyzed its association with ICT use. Subgroup analyses were stratified according to gender, years of education, and living arrangements.

**Results:**

Higher ICT use was not associated with frailty after adjusting for covariates (odds ratio [OR]: 0.53; 95%CI 0.39–0.73). Similar associations were found in the sub-groups of women (OR 0.45, 95%CI 0.30–0.66), <13 years of education (OR 0.48, 95%CI 0.34–0.67), living alone (OR 0.46, 95%CI 0.27–0.79), and living together (OR 0.57, 95%CI 0.38–0.85). No association existed between using ICT and frailty in the sub-groups of men and ≥13 years of education.

**Conclusions and implications:**

Higher ICT use is associated with the absence of frailty in individuals 75 years and older. Such benefits may be particularly pronounced in women, those with lower levels of education, and older adults living alone or with others.

**Supplementary Information:**

The online version contains supplementary material available at 10.1007/s41999-024-00979-y.

## Key summary points

**Aim:**

To investigate whether ICT use is associated with frailty among community-dwelling older people aged 75 and older.

**Findings:**

Higher ICT use was linked to absence of frailty among persons aged 75 years and older, and comparable findings were observed when stratified by gender, years of education, and living arrangements.

**Message:**

This cross-sectional study found that higher ICT use was associated with less frailty; ICT use and frailty are closely related and causal relationships need to be clarified in the future.

**Supplementary Information:**

The online version contains supplementary material available at 10.1007/s41999-024-00979-y.

## Introduction

Frailty, a state of increased vulnerability to stress due to reduced physiological reserves in old age, is a significant cause of adverse health events that include conditions requiring long-term care and hospitalization [1–3]. The prevalence of frailty among community-dwelling older adults worldwide is estimated to be 10.7% [4]. Older adults with frailty incur higher costs associated with care and healthcare than those without frailty [5, 6]. In particular, the prevalence of frailty and the costs associated with care and medical treatment are many times higher for those over 75 years of age than for younger people, leading to more problematic public health events [7]. Reducing the risk and prevalence of frailty is expected to play an essential role in extending the healthy life expectancy of older adults. In recent years, there has been a focus on using information and communication technologies (ICT) as a cost-effective way to manage frailty proactively. However, it has been noted that ease of use and technological acceptance are not yet in place in real-world settings [8].

The digital divide has recently been identified as the gap between those without access to computers and those with access to other ICT [9]. It has been reported that older people who frequently use computers and other familiar ICT devices have a lower risk of developing mild cognitive impairment [10]. The use of mobile devices, such as smartphones, has also been shown to be potentially effective in maintaining the quality of life [11]. As mobility declines with age, the frequency of face-to-face interactions and grocery shopping may decrease. With ICT, older adults can communicate through group chats and e-mails, and participate in online shopping activities. Moreover, because age-related chronic diseases increase the need for health knowledge and information about medications [12], ICT may be used to obtain helpful information related to health and medical conditions [13], thereby increasing health literacy. Hence, ICT may be associated with frailty and cognitive and physical function in older people.

However, studies examining the association between ICT use and frailty are limited and require validation [14]. Based on studies with large sample sizes, clarifying the relationship between ICT use and frailty is crucial. At the same time, the association needs to be analyzed separately according to the target population’s characteristics (e.g., sex and educational background), given the characteristics of frailty, which are assumed to be more common among old–old adults and women [4, 15, 16]. Therefore, this study aimed to examine the association between the ability to use ICT and frailty using data from the Integrated Longitudinal Studies on Aging in Japan (ILSA-J) [17], which integrates data from a representative cohort study in Japan. Gender, education, and living arrangement (living alone or with others) were stratified and validated. The results of this study provide fundamental insights into the importance of considering the use of ICT to prevent frailty.

## Methods

### Study design and data sources

The data source for this study was the ILSA-J, which aimed to establish Japan’s first platform for understanding changes in physical and mental function over time in Japanese community-dwelling older people aged 65 and over. The criteria for cohort studies to be included in the ILSA-J are that they must be conducted in Japan, include community-dwelling older adults aged 65 years or older, must be observational studies using standard measures, and have a published study design [17]. A total of 16 longitudinal cohort studies conducted in Japan were included in ILSA-J.

**Fig. 1:**
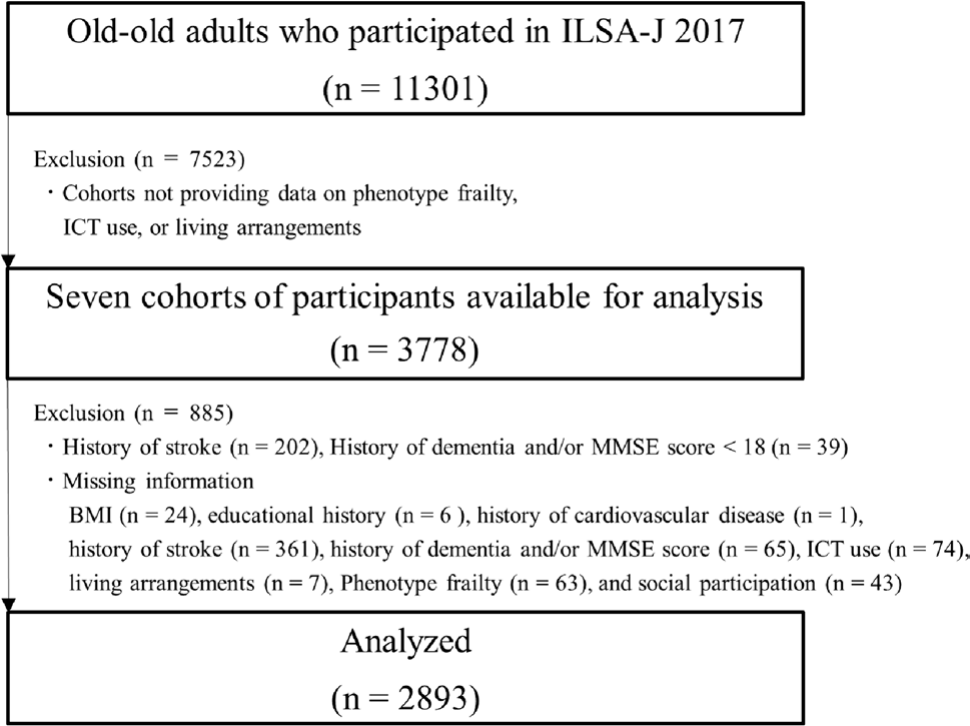
Flow chart for the inclusion and exclusion of participants in the study

Seven cohort studies that assessed frailty using the J-CHS based on the Fried criteria (i.e., slowness, weakness, exhaustion, low activity, and weight loss) [15], ability to use ICT, and basic characteristics for stratification in old–old adults aged 75 years and older were included in this analysis (Appendix Table 1). A flowchart of the participants is shown in Fig. 1. For the 3778 participants enrolled in the seven cohorts studied in 2017 (±1 year), participants with stroke (*n* = 202), dementia and/or Mini Mental State Examination [18] score <18 (*n* = 39), and missing data values (*n* = 644) were excluded. Finally, 2893 participants were included in the study. The ethics committee of the respective university or institute approved all cohort studies and the ILSA-J.

**Table 1:** Comparison of characteristics of individuals with and without frailty

	Overall *n* = 2893	Non-frailty *n* = 2685	Frailty *n* = 208	*p*-value
Age, mean ± SD (years)	79.6 ± 3.9	79.5 ± 3.8	81.9 ± 4.9	<0.001
Women, *n* (%)	1796 (62.1)	1666 (62.0)	130 (62.5)	0.897
Body mass index, mean ± SD (kg/m^2^)	22.8 ± 3.2	22.9 ± 3.2	22.3 ± 3.9	0.046
Living alone, *n* (%)	878 (30.3)	811 (30.2)	67 (32.2)	0.544
Education (less than 13 years), *n* (%)	2095 (72.4)	1928 (71.8)	167 (80.3)	0.008
Medical history, *n* (%)				
Hypertension	1388 (48.0)	1270 (47.3)	118 (56.7)	0.009
Diabetes mellitus	347 (12.0)	304 (11.3)	43 (20.7)	<0.001
Cardiovascular disease	414 (14.3)	378 (14.1)	36 (17.3)	0.200
Social participation, *n* (%)	2088 (72.2)	1969 (73.3)	119 (57.2)	<0.001
ICT use, *n* (%)				
Use of mobile phones	2395 (82.8)	2256 (84.0)	139 (66.8)	<0.001
Use of ATM	2276 (78.7)	2137 (79.6)	139 (66.8)	<0.001
Operation of video recorder	1729 (59.8)	1641 (61.1)	88 (42.3)	<0.001
Sending e-mail	1642 (56.8)	1561 (58.1)	81 (38.9)	<0.001
ICT users, *n* (%)	2332 (80.6)	2202 (82.0)	130 (62.5)	<0.001

*ICT* information and communication technologies, *SD* standard deviation

Significant *p*-values are indicated in bold

### Assessment of ICT use

We determined the ability to use ICT according to the four criteria for “technology usage” in previous studies: using a mobile phone, using an ATM, operating a video recorder, and sending e-mail [19, 20]. Participants self-administered each item on a “yes (able to do)” or “no (unable)” scale, with “yes” scored as one and “no” scored as 0. The four criteria defining ICT use were as follows: “Can you use a mobile phone?”, “Can you use an ATM?”, “Can you operate a video recorder such as a Blu-ray recorder or DVD player?”, “Can you send an e-mail using a mobile phone or computer?”. Higher scores indicated higher levels of ICT use. In this study, a total score of 1 or less, corresponding to a score in the bottom 20%, was defined as “ICT non-users” [21]. A score of 2 or higher was defined as “ICT users.”

### Assessment of frailty

Frailty status was determined according to the five criteria for physical frailty proposed in the Japanese version of the Cardiovascular Health Study (J-CHS) [3, 22], with slightly modified criteria: weight loss, slowness, weakness, exhaustion, and low activity, with the criteria for low activity differing slightly from the original definition. Weight loss was identified by a response of “yes” to the question, “Have you lost 2 kg or more in the past six months?” [23] Slowness was defined as normal walking speed <1.0 m/sec [24]. Weakness was identified based on the grip strength of the subject’s dominant hand (<28 kg for men and <18 kg for women [25]. Exhaustion was identified by a response of “yes” to the question, “In the last two weeks, have you felt tired for no reason?” [23] In five cohorts, low activity was identified by a response of “no” to both the following questions: “Do you engage in moderate levels of physical exercise or sports aimed at health?” and “Do you engage in low levels of physical exercise aimed at health?” [24] in one cohort: 1) Take regular walks, 2) Perform light gymnastics regularly, 3) Exercise regularly. In one cohort, defined as the lowest 20% of physical activity (METs/day) by sex, as measured by the Global Physical Activity Questionnaire [26]. Participants who responded positively to three or more criteria were considered frail.

### Sociodemographic variables and covariates

Previous studies reported that demographic characteristics, chronic disease status, and social participation are associated with frailty in older adults [4, 27, 28]. Hence, we included age, gender, body mass index (BMI), living alone, education, medical history, and social participation as covariates, based on previous studies. Demographic information including age (years), gender, BMI, living alone, education (<13 years, ≥13 years), history of hypertension, diabetes, cardiovascular disease, and social participation were evaluated. Those who answered “yes” to any of the following questions were defined as having social participation: “Do you participate in regional festivals or events?” “Do you participate in a neighborhood association or a residents’ association?” “Would you be able to assume a managerial position as an organizer in a residents’ association or group activity?” “Do you engage in charity or volunteer activities?” [19, 20, 29].

### Statistical analysis

Participant characteristics are expressed as mean and standard deviation for continuous variables and percentage (%) for categorical variables. The t-test and chi-square test were used for continuous and categorical variables, respectively, to compare the differences between the non-frailty and frailty groups. The association between ICT use and frailty was examined using multivariate logistic regression analysis, with ICT use as the independent variable and frailty as the dependent variable. The multivariate model included the following covariates: age, gender, BMI, living alone, education, medical history (hypertension, diabetes, and cardiovascular disease), and social participation. Similar stratified analyses were performed for gender (men or women), education (<13 or ≥13 years), and living arrangements (living alone or living with others). All analyses were performed using IBM SPSS Statistics 26.0 (IBM Japan, Tokyo, Japan). The level of statistical significance was set at *p* < 0.05.

## Results

The mean age of the participants was 79.6 ± 3.9 years and 62.1% were women (Table 1). Of the adults aged 75 years enrolled in this study, 208 (7.2%) were frail. The results of ICT use showed that 82.8% of the participants were using a mobile phone, 78.7% were using an ATM, 59.8% were operating a video recorder, and 56.8% were sending e-mails. In group comparisons, the frailty group was older (*p* < 0.001), had more than 13 years of education (*p* = 0.008), had more hypertension (*p* = 0.009) and diabetes (*p* < 0.001), had lower BMI (*p* = 0.046), and less social participation (*p* < 0.001) than the non-frailty group. The frail group had significantly fewer ICT users than the non-frail group (*p* < 0.001).

Multivariate logistic regression analysis (Table 2) adjusted for sociodemographic variables revealed ICT users had a significantly lower odds ratio of being frailty than non-ICT users [odds ratio (OR) 0.53, 95% confidence interval (CI) 0.39–0.73; *p* < 0.001].

**Table 2:** Logistic regression models of the association between ICT use and frailty (*n* = 2893)

Independent variable	Crude			Adjusted model		
	Wald	OR (95% CI)	*p*-value	Wald	OR (95% CI)	*p*-value
ICT non-users		1 (ref)			1 (ref)	
ICT users	43.96	0.37 (0.27–0.49)	<0.001	15.28	0.53 (0.39–0.73)	<0.001
Age				38.25	1.11 (1.07–1.15)	<0.001
Gender				0.08	1.05 (0.76–1.45)	0.772
Body mass index				5.58	0.95 (0.90–0.99)	0.018
Living alone				0.12	0.94 (0.68–1.31)	0.728
Education				2.51	1.35 (0.93–1.96)	0.113
Medical history						
Hypertension				2.87	1.30 (0.96–1.76)	0.090
Diabetes mellitus				15.35	2.11 (1.45–3.07)	<0.001
Cardiovascular disease				0.42	1.14 (0.77–1.69)	0.518
Social participation				13.62	0.57 (0.42–0.77)	<0.001

*95% CI* 95% confidence interval, *OR* odds ratio

Significant *p*-values are indicated in bold. Adjusted model: age, gender, body mass index, living alone, education, medical history (hypertension, diabetes mellitus, and cardiovascular disease), and social participation

Hosmer–Lemeshow goodness-of-fit test: *p* = 0.921

**Fig. 2:**
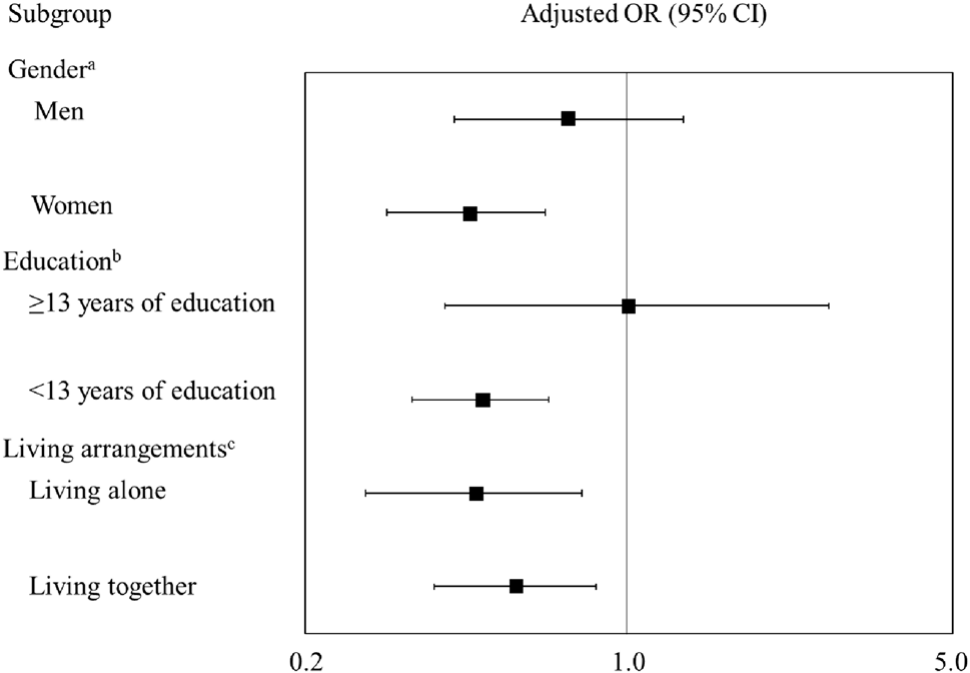
Subgroup analyses of the association between ICT use and frailty. Note: Adjusted OR of ICT users compared to ICT non-users (reference). ^a^Adjusted model: age, body mass index, living alone, education, medical history (hypertension, diabetes mellitus, and cardiovascular disease), and social participation. ^b^Adjusted model: age, sex, body mass index, living alone, medical history (hypertension, diabetes mellitus, and cardiovascular disease), and social participation. ^c^Adjusted model: age, sex, body mass index, education, medical history (hypertension, diabetes mellitus, and cardiovascular disease), and social participation. *95% CI* 95% confidence interval, *OR* odds ratio

The results of the stratified multivariate logistic regression analysis are shown in Fig. 2. Each stratum was defined according to sex, education, and living arrangement. ICT users had lower odds ratios for frailty in various strata, even after adjusting for age, gender, BMI, living arrangement, education, medical history, and social participation. Specifically, among subgroups of women (OR 0.45, 95%CI 0.30–0.66; *p* < 0.001), lower education (OR 0.48, 95%CI 0.34–0.67; *p* < 0.001), living alone (OR 0.46, 95%CI 0.27–0.79; *p* = 0.005), and living with others (OR 0.57, 95%CI 0.38–0.85; *p* = 0.005), ICT users showed lower odds ratios for frailty. No significant association existed between using ICT and frailty in men (OR 0.74, 95%CI 0.42–1.31; *p* = 0.298) and higher education (OR 1.04, 95%CI 0.40–2.70; *p* = 0.941).

## Discussion

This study examined the association between ICT use and frailty in Japanese people aged 75 years and older. The results showed that maintaining ICT use was associated with a lower odds ratio for frailty, after adjusting for confounding factors. The results were also similar in the subgroup analyses for women, those with fewer years of education, those living alone, and those living with others. These results are similar to those of previous studies that examined the association between ICT use and frailty using questionnaires [30]. However, the present study is novel because it assessed frailty using the gold standard J-CHS criteria and performed several stratified analyses.

The maintenance of ICT use has been reported to prevent cognitive decline [10]. It has been shown that cognitive function declines in old age in interaction with physical function [31, 32] and ICT use may positively affect both functions and may be protective of frailty. A previous study reported that decreased ICT use was associated with cognitive frailty, a coexisting condition of mild cognitive impairment, and a decline in physical function [21]. In addition, ICT use may improve social isolation and low ICT use is associated with depressive symptoms and poor subjective health [33, 34]. These findings suggest that the use of ICT may function as a protective factor against psychosomatic functional decline compared to not using ICT. The present study did not investigate the possible mechanisms underlying the association between the maintenance of ICT use and frailty; however, elucidation of these mechanisms is essential in the future.

The prevalence of frailty may differ according to demographics such as gender and years of education [35, 36]. In this study, differences in the results were also observed for several items, such as age and years of education. Moreover, we found an association between the maintenance of ICT use and lower odds ratios for frailty, even among women and populations with fewer years of education, which are generally assumed to have higher prevalence rates [24]. Studies on health-related ICT use indicate that the percentage of women who refuse to use ICT increases with age [37]. Psychosocial factors such as stigma and shame are thought to be involved in this background [38]. Some reports suggest that ICT use is associated with quality of life and health literacy [11, 39, 40] and that access to more information and interaction with others may help people maintain better health behaviors. Promoting ICT use among these groups may be effective; however, future studies are needed to verify this.

Stratified analysis revealed no association with frailty in men or in the population with more years of education. This result may be due to the higher overall use of ICT in this group than in the other groups. However, we cannot exclude the possibility of negative effects. ICT use as a potential stressor, often observed in younger and middle-aged people [41], has recently been found in some older people; the stress associated with ICT use has been described as technostress, with older people being particularly stressed by issues of privacy and complexity [42]. Old–old adults have more difficulty than other age groups in actively adapting to new functions such as ICT [43]. To promote the use of ICT, it is necessary to design and operate ICT systems that are easy for older people to use [44].

Because this study included a prominent cohort representative of Japan, it is assumed that the external validity of the results in the Japanese population is relatively high. Positive attitudes toward ICT use are related to cultural background, age, and educational level [45]. Additional validation is required to determine whether similar results can be obtained in other countries. Compared to previous studies, this study had a larger sample size among studies of populations aged 75 years or older and the criteria for determining frailty were more objective.

The limitations of this study include the slightly lower prevalence of frailty (7.2%) among participants and the fact that causal relationships are unknown due to the cross-sectional nature of the analysis. The relationship between ICT uses and frailty needs to be investigated bidirectionally through cohort studies and the effectiveness of promoting ICT use needs to be tested in intervention studies.

## Conclusions and implications

In conclusion, we found an association between ICT use and lower odds ratios for frailty among community-dwelling older adults aged 75 years and older. ICT use is closely related to frailty and causal relationships need to be clarified in the future.

### Supplementary Information

Below is the link to the electronic supplementary material.

Supplementary file1 (DOCX 30 KB)

## Data Availability

The data underlying this article cannot be shared publicly due to privacy or ethical restrictions.
