# Supplementary material for: Associations between information and communication technology use and frailty in community-dwelling old-old adults: results from the ILSA-J
Source: Eur Geriatr Med. 2024 May 9;15(3):621–7. doi: 10.1007/s41999-024-00979-y (PMC11329410; doi:10.1007/s41999-024-00979-y)
Supplement: Supplementary file 1 — Supplementary file1 (DOCX 30 KB) [file 41999_2024_979_MOESM1_ESM.docx]

Appendix table 1. Frailty criteria

| Cohort (Geographic location in Japan) | Weight loss | Slowness | Weakness | Exhaustion | Low activity |
| --- | --- | --- | --- | --- | --- |
| A (Mid-eastern part) | Unintentional weight loss ≥2-3 kg/6 months (Kihon Checklist #11) | Normal walking speed: <1.0 m/s (distance: 5 m） | Grip strength: men <28 kg, women <18 kg | Constant tiredness in the past 2 weeks (Kihon Checklist #25) | 1) Low levels of physical exercise <1 day/week  2) Regular physical activities <1 day/week  Applicable to both questions |
| B (Capital area) | Unintentional weight loss ≥2-3 kg/6 months (Kihon Checklist #11) | Normal walking speed: <1.0 m/s (distance: 5 m） | Grip strength: women <18 kg (only women data) | Constant tiredness in the past 2 weeks (Kihon Checklist #25) | 1) Take regular walks  2) Perform light gymnastics regularly  3) Exercise regularly  Not applicable to all questions |
| C (Capital area) | Unintentional weight loss ≥2-3 kg/6 months (Kihon Checklist #11) | Normal walking speed: <1.0 m/s (distance: 5 m） | Grip strength: men <28 kg, women <18 kg | Constant tiredness in the past 2 weeks (Kihon Checklist #25) | 1) Low levels of physical exercise <1 day/week  2) Regular physical activities <1 day/week  Applicable to both questions |
| D (Capital area) | Unintentional weight loss ≥2-3 kg/6 months (Kihon Checklist #11) | Normal walking speed: <1.0 m/s (distance: 5 m） | Grip strength: men <28 kg, women <18 kg | Constant tiredness in the past 2 weeks (Kihon Checklist #25) | 1) Low levels of physical exercise <1 day/week  2) Regular physical activities <1 day/week  Applicable to both questions |
| E (Suburbs of capital area) | Unintentional weight loss ≥2-3 kg/6 months (Kihon Checklist #11) | Normal walking speed: <1.0 m/s (distance: 5 m） | Grip strength: men <28 kg, women <18 kg | Constant tiredness in the past 2 weeks (Kihon Checklist #25) | The lower 20 % of physical activity (METs/day) by gender (Global Physical Activity Questionnaire) |
| F (Southwestern part) | Unintentional weight loss ≥2-3 kg/6 months (Kihon Checklist #11) | Normal walking speed: <1.0 m/s (distance: 5 m） | Grip strength: men <28 kg, women <18 kg | Constant tiredness in the past 2 weeks (Kihon Checklist #25) | 1) Low levels of physical exercise <1 day/week  2) Regular physical activities <1 day/week  Applicable to both questions |
| G (Southern part) | Unintentional weight loss ≥2-3 kg/6 months (Kihon Checklist #11) | Normal walking speed: <1.0 m/s (distance: 10 m） | Grip strength: men <28 kg, women <18 kg | Constant tiredness in the past 2 weeks (Kihon Checklist #25) | 1) Low levels of physical exercise <1 day/week  2) Regular physical activities <1 day/week  Applicable to both questions |
